# Marine n-3 Polyunsaturated Fatty Acids and Cellular Senescence Markers in Incident Kidney Transplant Recipients: The Omega-3 Fatty Acids in Renal Transplantation (ORENTRA) Randomized Clinical Trial

**DOI:** 10.1016/j.xkme.2021.07.010

**Published:** 2021-10-04

**Authors:** Joe Chan, Ivar A. Eide, Tone M. Tannæs, Bård Waldum-Grevbo, Trond Jenssen, My Svensson

**Affiliations:** 1 Department of Renal Medicine, Akershus University Hospital, Lørenskog; 2 Institute of Clinical Medicine, Faculty of Medicine, University of Oslo, Oslo; 3 Department of Transplantation Medicine, Oslo University Hospital Rikshospitalet, Oslo; 4 Department of Clinical Molecular Biology (EpiGen), Division of Medicine, Akershus University Hospital and University of Oslo, Lørenskog; 5 Department of Nephrology, Oslo University Hospital Ullevål, Oslo, Norway

**Keywords:** Assay validation, cellular senescence, fatty acid, interleukin, intervention, kidney transplantation, macrophage inflammatory protein, matrix metalloproteinase, monocyte chemoattractant protein, multiplex, omega-3, senescence-associated secretory phenotype, transforming growth factor, tumor necrosis factor

## Abstract

**Rationale & Objective:**

Deterioration of kidney graft function is associated with accelerated cellular senescence. Marine n-3 polyunsaturated fatty acids (PUFAs) have favorable properties that may counteract cellular senescence development and damage caused by the senescence-associated secretory phenotype (SASP) secretome. Our objective was to investigate the potential effects of marine n-3 PUFA supplementation on the SASP secretome in kidney transplant recipients.

**Study Design:**

Exploratory substudy of the Omega-3 Fatty Acids in Renal Transplantation trial.

**Setting & Participants:**

Adult kidney transplant recipients with a functional kidney graft (defined as having an estimated glomerular filtration rate of >30 mL/min/1.73 m^2^) 8 weeks after engraftment were included in this study conducted in Norway.

**Analytical Approach:**

The intervention consisted of 2.6 g of a marine n-3 PUFA or olive oil (placebo) daily for 44 weeks. The outcome was a predefined panel of SASP components in the plasma and urine.

**Results:**

A total of 132 patients were enrolled in the Omega-3 Fatty Acids in Renal Transplantation trial, and 66 patients were allocated to receive either the study drug or placebo. The intervention with the marine n-3 PUFA was associated with reduced plasma levels of granulocyte colony-stimulating factor, interleukin 1α, macrophage inflammatory protein 1α, matrix metalloproteinase (MMP)-1, and MMP-13 compared with the intervention in the control group.

**Limitations:**

Post hoc analysis.

**Conclusions:**

The results suggest that marine n-3 PUFA supplementation has mitigating effects on the plasma SASP components granulocyte colony-stimulating factor, interleukin 1α, macrophage inflammatory protein 1α, MMP-1, and MMP-13 in kidney transplant recipients. Future studies with kidney transplant recipients in maintenance phase, combined with an evaluation of cellular senescence markers in kidney transplant biopsies, are needed to further elucidate the potential antisenescent effect of marine n-3 PUFAs. This trial is registered as NCT01744067.

**Plain-Language Summary**

Deterioration of kidney transplant function is associated with accelerated cellular senescence. In this exploratory sub-study of the randomized controlled trial ORENTRA, we investigated the potential anti-senescent effects of marine n-3 fatty acid supplementation by analyzing plasma and urine samples from 132 incident kidney transplant recipients. We looked for effects on components of the senescence-associated secretory phenotype, and we found that intervention with marine n-3 fatty acids was associated with lower plasma levels of granulocyte colony-stimulating factor, interleukin 1α, macrophage inflammatory protein 1α, matrix metalloproteinase (MMP)-1 and MMP-13. Future studies with kidney transplant recipients in maintenance phase combined with evaluation of senescence markers in kidney transplant biopsies are encouraged to further elucidate the potential anti-senescent effect of marine n-3 fatty acids.

Cellular senescence, the permanent arrest of cell growth, is essential for embryonic organ development, tissue repair, and cancer protection.^1^ In these settings, cellular senescence is a tightly regulated process with specific homeostatic functions. However, cellular senescence is also associated with progressive organ dysfunction in patients with aging and age-related diseases, such as atherosclerosis, diabetes, cancer, and chronic kidney disease.^1^^,^^2^ The transition of a cell to a senescent state may be induced by various stressors, including telomere attrition, DNA damage, reactive oxygen species, oncogene activation, epigenetic stress, mitochondrial dysfunction, and inflammation.^3^ Senescent cells are characterized by the expression of cyclin-dependent kinase inhibitor 2A (p16^INK4a^), increased senescence-associated β-galactosidase activity, resistance to apoptosis, and a senescence-associated secretory phenotype (SASP).^2^ The components of SASP include inflammatory cytokines, growth factors, chemokines, proteases, and other reactive molecules, which lead to chronic sterile inflammation, affecting neighboring cells and driving further senescence development.^4^^,^^5^

Kidney transplant recipients with grafts with interstitial fibrosis and tubular atrophy^6^ and those with chronic allograft rejection^7^ express higher levels of p16^INK4a^ than what is expected for their age, consistent with accumulation of senescent cells. High p16^INK4a^ expression in kidney graft biopsies at the time of engraftment is a strong predictor of an adverse long-term transplant outcome.^8^, ^9^, ^10^ Immunosuppressive drugs, such as calcineurin inhibitors, may induce cellular senescence and hamper the clearance of senescent cells,^11^, ^12^, ^13^ causing further assault by SASP factors on adjacent tissue. In murine studies, p16^INK4a^ deletion has been shown to prevent kidney tubulointerstitial injury, alleviate senescence, inhibit the secretion of SASP factors, and promote proliferation,^14^ which may explain the reduced interstitial fibrosis and tubular atrophy as well as improved graft survival.^15^ Thus, to preserve long-term kidney graft function, a therapeutic option that targets cellular senescence is desirable. However, the development of drugs that specifically remove senescent cells (senolytics) is still in an early phase.^16^

Marine n-3 polyunsaturated fatty acids (PUFAs) have well-described favorable effects that may inhibit SASP secretome production^17^ and, hence, possibly possess a senostatic function. In cell culture studies, marine n-3 PUFAs inhibit tumor necrosis factor-induced senescence^18^ and attenuate oxidative stress-induced DNA damage^19^ in endothelial cells. In addition, 1 prospective cohort study found an association between high levels of a marine n-3 PUFA and low rates of telomere shortening in patients with coronary heart disease.^20^ Consistent with this finding, 2 intervention studies showed that marine n-3 PUFA supplementation attenuated telomere shortening.^21^^,^^22^

Our study group has previously demonstrated that in kidney transplant recipients, the plasma levels of marine n-3 PUFAs are inversely correlated with well-known components of the SASP secretome, such as soluble tumor necrosis factor receptor 1 and interleukin (IL) 6.^4^^,^^23^ The recently published randomized controlled trial “Omega-3 Fatty Acids in Renal Transplantation (ORENTRA)” showed reductions in plasma high-sensitivity C-reactive protein levels and improvement in endothelial function after 44 weeks of high-dose n-3 PUFA supplementation.^24^ These findings could be related to potential effects on cellular senescence and SASP components. This is an exploratory substudy of the ORENTRA trial, and the aim was to investigate the potential effects of marine n-3 PUFA supplementation on the SASP secretome in kidney transplant recipients.

## Methods

### Study Design

Details regarding the design of the ORENTRA trial have been previously described in detail.^24^ In brief, it was a randomized, double-blind, placebo-controlled trial with a cohort consisting of Norwegian kidney transplant recipients aged 18 years or above with a functional kidney graft (defined as having an estimated glomerular filtration rate of >30 mL/min/1.73 m^2^) at the time of inclusion. The trial duration was 44 weeks, with baseline at 8 weeks after engraftment and end of the study at 1 year after transplantation. Patients were randomly assigned to receive either treatment with the marine n-3 PUFA ethyl ester (460 mg/g of eicosapentaenoic acid and 380 mg/g of docosahexaenoic acid given as 3 capsules of 1 g, ≈2.6 g effective dose of eicosapentaenoic acid and docosahexaenoic acid per day) or a placebo (extra virgin olive oil, 3 capsules of 1 g per day). The patients were otherwise treated according to the local standard immunosuppression protocol, with prednisolone, tacrolimus, and mycophenolate. The primary endpoint was a change in the measured glomerular filtration rate (mGFR) during follow-up. The key secondary endpoints included measurement of the degree of kidney graft fibrosis and proteinuria, level of high-sensitivity C-reactive protein and lipoproteins, and extent of flow-mediated dilation as a measure of endothelial function. Written informed consent for participation was obtained from all patients. The study was approved by the Regional Committees for Medical and Health Research Ethics in Norway (REK 2012/1419) and The Norwegian Medicines Agency. It was performed in accordance with the Declaration of Helsinki and the principles of Good Clinical Practice.

### Selection of SASP Components

Based on the existing research, we selected SASP components that are found across most cell types and those described in relation to kidney disease.^1^^,^^4^^,^^25^, ^26^, ^27^, ^28^ In total, we included 19 components: granulocyte colony-stimulating factor (G-CSF); granulocyte-macrophage colony-stimulating factor; growth-related oncogene α; IL-6; IL-8; IL-1α; IL-1β; macrophage inflammatory protein (MIP) 1α; matrix metalloproteinase (MMP)-1; MMP-2; MMP-3; MMP-9; MMP-13; monocyte chemoattractant proteins 1, 2, and 3; plasminogen activator inhibitor-1; transforming growth factor β1 (TGF-β1); and tumor necrosis factor α. The levels of all the components were measured in the plasma and urine, except for TGF-β1. The measurement of the TGF-β1 level in the plasma is complex and prone to confounding factors if meticulous precautions are not taken while drawing blood and during plasma preparation.^29^ Therefore, the TGF-β1 level was only measured in the urine.

### Laboratory Assessments

The plasma and urine samples were collected with the patients in a fasting state in the morning during baseline and end-of-study visits and immediately stored at −70 °C to −80 °C. Individual fatty acid levels in the total plasma phospholipid extract were determined using gas chromatography and quantified as the weight percentage (wt%) of the total plasma phospholipid fatty acid level.^30^ The marine n-3 PUFA level was defined as the sum of the levels of eicosapentaenoic acid and docosahexaenoic acid.

The SASP components were measured using customized multiplex immunoassay panels (ProcartaPlex, ThermoFisher Scientific or Invitrogen) on the Bio-Plex 200 system. All markers were analyzed according to the manufacturer’s instructions in duplicate. A coefficient of variation <30% between the duplicate analyses was considered acceptable per the manufacturer’s recommendation.

Because of the effect of matrix interference in the analysis of urinary biomarkers,^31^ a pilot study was conducted in advance using urine samples from 4 patients randomly selected from the study cohort. The pilot study’s results are shown in Table S1. Diluting the samples in ratios of 1:10 and 1:20 resulted in the highest measurement values for most of the variables included. For the main analysis, a dilution ratio of 1:10 was chosen because a higher dilution for variables with low concentrations could have resulted in concentrations that could have been below the quantification limit of the assay. All the urine measurement values were normalized to urine creatinine concentration.

### Statistical Analysis

The sample size was based on the primary endpoint, a change in mGFR, of the parent study (ORENTRA trial). The study variables were assessed for normality using the Kolmogorov-Smirnov and Shapiro-Wilk tests as well as normality plots. The data were reported as mean ± standard deviation for values with a normal distribution and as median (25th, 75th percentiles) for values with a nonnormal distribution. Differences in postinterventional changes between the study groups were evaluated using analysis of covariance or the Quade’s test when assumptions for the analysis of covariance were not met, with adjustment for baseline values. Some variables had sample concentrations below the quantification limit of the assay. In these samples, we assigned a value lower by 1 at the last decimal place of the lowest measured value of the corresponding variable before the statistical analysis. Associations between the plasma and the corresponding urine variables were assessed using the Pearson’s correlation coefficient (*r*) for the baseline and end-of-study values. The same method was used to evaluate associations between changes in the levels of significant SASP components and mGFR. All the tests were 2 sided and assessed at a *P* value of <0.05 level of significance. The statistical analysis was performed using Statistical Product and Service Solutions, version 26.

## Results

### Study Participants, Safety, and Study Drug Adherence

**Figure 1:**
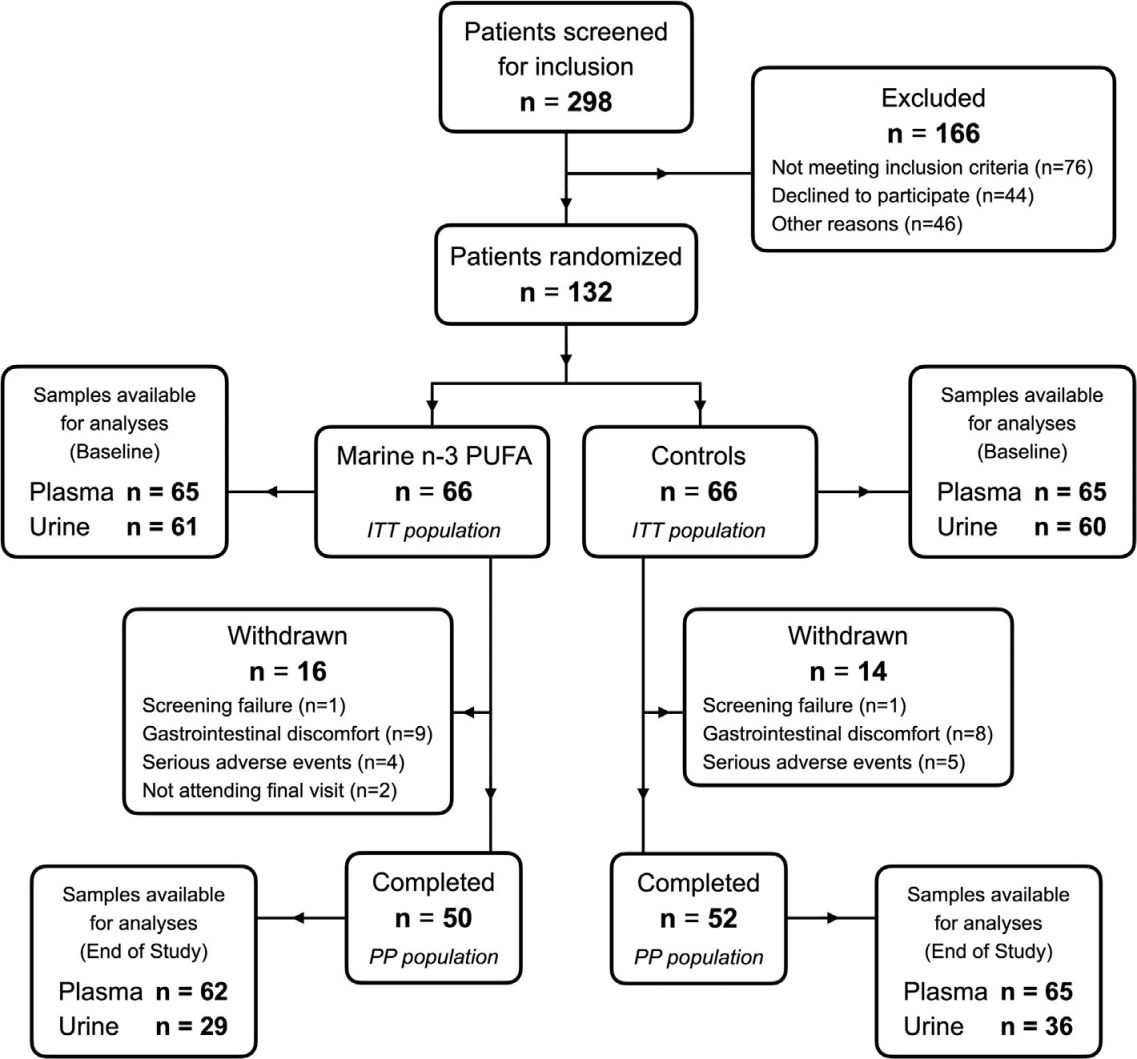
Study flowchart presenting patient screening, randomization, reasons for withdrawal from the trial, and the number of plasma and urine samples available from each group at baseline and at the end of the study. Abbreviations: ITT, intention to treat; PP, per protocol; PUFA, polyunsaturated fatty acid.

A total of 132 patients were enrolled in the ORENTRA trial, and 66 patients were allocated to receive either the study drug or the placebo (Fig 1). The baseline patient characteristics are presented in Table 1. There were 218 and 240 adverse events in the marine n-3 PUFA and control groups, respectively, during follow-up. Gastrointestinal side effects were the most frequent type of adverse event and the most common reason for withdrawal from the trial in both the groups. Fifteen episodes of acute rejections occurred among 11 patients in the marine n-3 PUFA group, and 16 episodes of acute rejections occurred among 14 patients in the control group. There was 1 death due to cancer in the marine n-3 PUFA group, and there was 1 case of graft failure in the control group. Tacrolimus trough levels at the end of the study were the same between the 2 groups. Study drug adherence was evaluated using the plasma marine n-3 PUFA levels, which increased by 4.0 ± 2.7 wt% in the marine n-3 PUFA group and remained unchanged in the control group at the end of the study.

**Table 1:** Baseline Patient Characteristics

	Marine n-3 PUFA Group	Control Group
Number of patients	66	66
Recipient age, y	52.8 ± 13.5	54.1 ± 14.2
Recipient sex, female	19 (28.8%)	15 (22.7%)
Marine n-3 PUFA, wt%	6.4 ± 2.2	6.3 ± 2.1
Eicosapentaenoic acid, wt%	1.8 ± 1.1	1.8 ± 1.1
Docosahexaenoic acid, wt%	4.6 ± 1.3	4.4 ± 1.3
Alpha linolenic acid, wt%	0.3 ± 0.1	0.3 ± 0.1
Linoleic acid, wt%	23.6 ± 3.0	24.2 ± 3.6
Arachidonic acid level, wt%	8.5 ± 1.8	8.5 ± 1.5
eGFR, mL/min/1.73 m^2^	70.7 ± 21.1	67.1 ± 21.8
mGFR, mL/min/1.73 m^2^	56.2 ± 15.3	54.9 ± 16.8
Body mass index, kg/m^2^	25.7 ± 3.8	26.3 ± 4.0
Fasting plasma glucose, mg/dL	102.0 ± 16.2	98.2 ± 11.8
p-Albumin, g/L	42.1 ± 2.8	42.7 ± 2.9
hsCRP, mg/L	4.6 ± 10.2	3.2 ± 4.3
Primary kidney disease		
Diabetes nephropathy	7 (10.6%)	10 (15.2%)
Hypertensive nephropathy	15 (22.7%)	16 (24.2%)
Glomerulonephritis	22 (33.3%)	18 (27.3%)
Other kidney diseases	22 (33.3%)	22 (33.3%)
Pretransplantation disease		
Hypertension	52 (78.8%)	42 (63.6%)
Diabetes mellitus	9 (13.6%)	13 (19.7%)
Coronary disease	8 (12.1%)	8 (12.1%)
Cancer	5 (7.6%)	9 (13.6%)
Systolic blood pressure, mm Hg	132 ± 14	135 ± 17
Diastolic blood pressure, mm Hg	81 ± 11	82 ± 9
Smoking habits		
Daily smoker	10 (15.2%)	10 (15.2%)
Nondaily smoker	2 (3.0%)	1 (1.5%)
Former heavy smoker	4 (6.1%)	7 (10.6%)
Former light smoker	21 (31.8 %)	22 (33.3%)
Lifelong nonsmoker	29 (43.9%)	26 (39.4%)

*Note*: Selected baseline patient characteristics are presented as mean ± standard deviation for continuous variables and percentage for categorical data. The eGFR data were calculated using the CKD-EPI (Chronic Kidney Disease Epidemiology Collaboration) formula.

Abbreviations: eGFR, estimated glomerular filtration rate; hsCRP, high-sensitivity C-reactive protein; mGFR, measured glomerular filtration rate; PUFA, polyunsaturated fatty acid.

### Outcomes of the Parent Trial

The primary finding was that there was no significant difference in mGFR in the marine n-3 PUFA group compared with that in the controls during 44 weeks of follow-up.^24^ In prespecified secondary endpoints, the plasma triglyceride and high-sensitivity C-reactive protein levels were significantly reduced and endothelial function was improved in the marine n-3 PUFA group. In a per-protocol population, kidney graft biopsies of the control group showed an increase in interstitial fibrosis percentage score, whereas it remained stable in the marine n-3 PUFA group.^24^

### Plasma Analyses

The baseline and end-of-study values of the measured SASP components in the plasma are presented in Table 2. A high percentage (40%-64%) of the plasma samples had concentrations that were below the detection limit of the assay for G-CSF, granulocyte-macrophage colony-stimulating factor, growth-related oncogene α, IL-1a, and IL-1β, and most of the measurable values of these markers were in the lower range of the standard curve.

**Table 2:** Study Variables at Baseline and at the End of the Study

	Marine n-3 PUFA Group				Control Group			
	n	Baseline	n	End of Study	n	Baseline	n	End of Study
Plasma G-CSF	65	0.34 (0.34-11.11)	62	0.34 (0.34-26.58)	65	0.34 (0.34-13.83)	65	14.00 (0.34-33.24)
Plasma GM-CSF	65	0.27 (0.26-7.63)	62	3.26 (0.26-14.32)	65	3.44 (0.26- 10.42)	65	7.06 (0.26-19.51)
Plasma GROα	65	0.91 (0.91-3.39)	62	0.91 (0.91-12.12)	65	0.91 (0.91-3.45)	65	0.91 (0.91-12.36)
Plasma IL-1α	65	0.00 (0.00-0.13)	62	0.00 (0.00-0.38)	65	0.00 (0.00-0.07)	65	0.00 (0.00-0.42)
Plasma IL-1β	65	0.09 (0.09-1.32)	62	0.64 (0.09-4.86)	65	1.01 (0.09-4.20)	65	2.77 (0.09-7.55)
Plasma IL-6	65	10.50 (4.35-16.32)	63	11.36 (5.85-22.28)	65	12.94 (6.79-21.77)	65	18.67 (8.63-29.39)
Plasma IL-8	65	5.40 (2.85-7.35)	62	5.96 (3.23-9.42)	65	5.76 (3.00-9.33)	65	7.09 (4.36-12.21)
Plasma MCP-1	65	128.39 (102.44-177.74)	62	113.28 (85.04-146.10)	65	139.51 (100.42-187.88)	65	119.73 (90.96-167.61)
Plasma MCP-2	65	22.70 (15.92-33.52)	62	23.23 (15.38-32.06)	65	26.13 (20.11-34.14)	65	28.98 (19.86-35.63)
Plasma MCP-3	65	77.87 (52.81-133.48)	62	116.67 (65.70-160.03)	65	101.19 (57.00-167.64)	65	152.78 (80.61-208.72)
Plasma MIP-1α	65	4.53 (0.05-11.87)	62	3.65 (0.05-19.32)	65	8.37 (1.78-20.52)	64	13.30 (4.95-28.73)
Plasma MMP-1	65	12.55 (6.51-22.19)	62	11.04 (6.65-20.02)	65	14.70 (4.92-25.44)	65	16.64 (9.15-29.62)
Plasma MMP-2	65	22,477.83 (17,447.48-27,865.26)	62	23,692.58 (18,501.92-31,784.69)	65	22,758.34 (18,209.68-31,517.06)	65	21,754.19 (17,952.58-31,968.48)
Plasma MMP-3	65	10,409.09 (5,605.88-16,677.54)	62	7,761.87 (4,952.31-12,906.81)	65	10,271.25 (6,702.03-20,735.08)	65	8,754.88 (4,665.35-14,710.41)
Plasma MMP-9	65	2,739.16 (1,526.92-4,078.28)	62	2,352.77 (1,265.70-3,709.61)	65	2,391.62 (1,577.94-3,305.78)	65	2,908.54 (1,721.23-4,367.60)
Plasma MMP-13	65	15.47 (6.86-32.17)	62	15.82 (4.57-36.89)	65	21.61 (11.45-32.28)	65	24.03 (14.56-46.40)
Plasma PAI-1	65	6,739.97 (5,403.22-9,020.59)	62	6,134.83 (4,256.72-7,945.50)	65	7,325.75 (5,482.58-9,454.51)	65	6,351.12 (4,783.31-8,396.98)
Plasma TNFα	65	15.29 (7.60-25.81)	62	21.11 (9.83-39.12)	65	23.12 (11.03-33.91)	65	30.86 (17.02-46.55)
Urine G-CSF	61	182.58 (100.21-301.08)	29	145.57 (71.20-209.83)	60	205.42 (123.24-307.74)	36	113.84 (44.51-213.23)
Urine IL-6	61	273.22 (191.04-349.46)	29	185.14 (142.67-229.14)	60	230.73 (177.30-295.88)	36	201.83 (143.80-279.26)
Urine IL-8	61	35.09 (11.79-92.11)	29	12.48 (1.40-72.75)	60	37.63 (11.40-78.47)	36	33.60 (2.92-72.15)
Urine MCP-1	61	2,345.13 (1,514.55-3,415.47)	29	900.43 (446.07-1,669.75)	60	1,630.37 (1,049.67- 2,468.70)	36	703.95 (465.85-1,774.79)
Urine MMP-3	61	624.65 (188.10-1,226.38)	29	210.11 (8.39-599.39)	60	929.70 (268.84-1,534.06)	36	124.84 (0.26-765.77)
Urine MMP-9	61	103.60 (59.45-183.17)	29	13.89 (4.07-167.88)	60	105.07 (44.02-254.05)	36	51.26 (12.67-194.48)
Urine TGF-β1	61	5.09 (0.87-16.82)	29	0.89 (0.24-7.05)	60	5.63 (0.67-21.68)	36	1.56 (0.31-13.32)

*Note*: Study variables are shown as medians (25th and 75th percentiles), plasma samples are in pg/mL, and urine samples are in ng/g creatinine.

Abbreviations: G-CSF, granulocyte colony-stimulating factor; GM-CSF, granulocyte-macrophage colony-stimulating factor; GROα, growth-regulated oncogene α; IL, interleukin; MCP, monocyte chemoattractant protein; MIP-1α, macrophage inflammatory protein 1α; MMP, matrix metalloproteinase; PAI-1, plasminogen activator inhibitor-1; PUFA, polyunsaturated fatty acid; TGF-β1, transforming growth factor β1; TNFα, tumor necrosis factor α.

### Urine Analyses

A relatively large number of urine samples were missing from both the study groups at the end of the study. This was partly because of exclusion of samples with signs of an ongoing urinary tract infection. Others were lost because of laboratory errors. Of all the SASP components analyzed, only IL-6, IL-8, G-CSF, monocyte chemoattractant protein 1, MMP-3, MMP-9, and TGF-β1 could be reliably measured in all the urine samples (Table 2). The remaining components either had concentrations below the quantification limit of the assay or had mostly estimated values below the range of the standard curve. Therefore, these components were excluded from the final statistical analysis.

### Treatment Effect

Postintervention changes in both the study groups are presented in Table 3. The marine n-3 PUFA group had significantly lower levels of G-CSF, IL-1α, MIP-1α, MMP-1, and MMP-13 in the plasma. There were no significant differences in the urinary SASP components between the 2 study groups.

**Table 3:** Postintervention Changes of the SASP Secretome

	Marine n-3 PUFA Group	Control Group	*P*
Plasma MMP-13	0.00 (−5.11 to 4.86)	6.14 (−1.20 to 15.84)	0.003
Plasma MMP-1	−0.47 (−7.51 to 5.70)	1.71 (−1.84 to 8.95)	0.007
Plasma MIP-1α	0.00 (−2.28 to 3.43)	1.93 (−0.73 to 8.72)	0.04
Plasma IL-1α	0.00 (0.00 to 0.06)	0.00 (0.00 to 0.26)	0.04
Plasma G-CSF	0.00 (0.00 to 6.22)	0.70 (0.00 to 18.82)	0.04
Plasma IL-6	1.12 (−2.26 to 7.25)	4.13 (−2.57 to 11.99)	0.06
Plasma TNFα	4.53 (−1.17 to 15.22)	8.47 (−0.03 to 18.98)	0.11
Plasma MCP-2	−2.32 (−6.85 to 3.34)	0.28 (−6.43 to 6.21)	0.15
Plasma IL-8	0.75 (−1.25 to 2.38)	0.96 (−0.43 to 3.28)	0.20
Plasma IL-1β	0.00 (0.00 to 1.56)	0.58 (0.00 to 4.82)	0.24
Plasma MMP-9	−148.57 (−1,226.76 to 956.81)	362.33 (−730.14 to 1,574.04)	0.32
Plasma MMP-3	−1,667.16 (−5,481.53 to −95.62)	−1,668.29 (−6,062.09 to −134.82)	0.33
Plasma MMP-2	1,091.31 (−2,366.65 to 4,695.02)	−724.09 (−3,826.00 to 3,126.47)	0.35
Plasma GM-CSF	0.00 (0.00 to 5.69)	1.59 (0.00 to 8.51)	0.37
Plasma MCP-3	22.74 (0.00 to 60.58)	41.09 (−10.86 to 88.58)	0.40
Plasma MCP-1	−6.57 (−51.73 to 13.38)	−15.06 (−44.65 to 10.09)	0.41
Plasma GROα	0.00 (0.00 to 3.76)	0.00 (0.00 to 4.98)	0.57
Plasma PAI-1	−764.77 ± 1,781.45	−688.53 ± 1,917.63	0.62
Urine TGF-β1	−0.14 (−6.06 to 3.12)	−2.99 (−19.22 to −0.03)	0.16
Urine MMP-3	−272.68 (−641.32 to 6.58)	−477.74 (−1,069.21 to −201.19)	0.32
Urine G-CSF	−33.03 ± 150.80	−90.59 ± 172.55	0.53
Urine IL-6	−53.17 ± 122.33	−34.07 ± 131.89	0.55
Urine MCP-1	−1,258.12 (−2,037.54 to −556.61)	−732.77 (−1,210.56 to −333.36)	0.60
Urine IL-8	−18.43 (−48.16 to −0.38)	−2.46 (−31.09 to 17.26)	0.75
Urine MMP-9	−57.52 (−138.96 to −10.39)	−53.75 (−195.66 to −8.78)	0.96

*Note*: Postintervention changes are shown as means ± standard deviations for values with a normal distribution and as medians (25^th^ and 75^th^ percentiles) for values with a nonnormal distribution. Differences between the study groups were evaluated using analysis of covariance or Quade’s test when assumptions for analysis of covariance were not met, both with adjustments for baseline values. Plasma samples are shown in pg/mL, and urine samples are in ng/g creatinine.

Abbreviations: G-CSF, granulocyte colony-stimulating factor; GM-CSF, granulocyte-macrophage colony-stimulating factor; GROα, growth-regulated oncogene α; IL, interleukin; MCP, monocyte chemoattractant protein; MIP-1α, macrophage inflammatory protein 1α; MMP, matrix metalloproteinase; PAI-1, plasminogen activator inhibitor-1; PUFA, polyunsaturated fatty acid; SASP, senescence-associated secretory phenotype; TGF-β1, transforming growth factor β1; TNFα, tumor necrosis factor α.

### Correlation Analyses

No significant correlations were found between the plasma and urine values at the baseline and the end of the study (Tables S2 and S3). No associations were found between changes in the levels of significant SASP components, mGFR, and interstitial fibrosis percentage scores, of kidney graft biopsies (Table S4).

## Discussion

To our knowledge, this is the first study that focused on the potential effects of marine n-3 PUFAs on the SASP secretome in kidney transplant recipients. We showed that intervention with 2.6 g of marine n-3 PUFAs per day was associated with reduced plasma levels of G-CSF, IL-1α, MIP-1α, MMP-1, and MMP-13 compared with the intervention in the control group.

Kidneys accumulate senescent cells with age.^7^ Well-known risk factors for chronic kidney disease, such as type 2 diabetes, hypertension, and glomerular diseases, may all cause accelerated cellular senescence,^32^, ^33^, ^34^ which in turn contribute to disease progression through the SASP secretome. In addition, cellular senescence is associated with interstitial fibrosis and tubular atrophy as well as chronic allograft nephropathy in kidney transplant recipients.^6^, ^7^, ^8^, ^9^, ^10^, ^11^, ^12^, ^13^ Although the elimination of senescent cells has shown promising results in animal studies,^14^^,^^15^ effective senolytic agents are still in an early stage of development.^16^

One possible therapeutic option for cellular senescence is marine n-3 PUFAs, which have anti-inflammatory and other beneficial properties^17^ that may counteract the development and effects of senescence and SASP. Major inflammatory cytokines of the SASP secretome can be induced by activation of nuclear factor kappa B.^35^ Previous studies have shown that marine n-3 PUFAs can decrease nuclear factor kappa B activation, possibly through a peroxisome proliferator activated receptor γ, with corresponding lower levels of inflammatory cytokines.^36^ Marine n-3 PUFA supplementation also increases the production of resolvins, protectins, and maresins, all of which have inflammation-resolving effects, and suppresses inflammatory cytokine production.^37^ Oxidative stress can induce cellular senescence, and marine n-3 PUFA supplementation may protect against the formation of reactive oxygen species.^22^

In the ORENTRA trial, the marine n-3 PUFA group had improved endothelial function and reduced high-sensitivity C-reactive protein levels during follow-up. In the per-protocol analysis, marine n-3 PUFA supplementation also prevented the development of graft fibrosis.^24^ This is in line with the findings of the present study; G-CSF, IL-1α, and MIP-1α are known proinflammatory cytokines^4^^,^^38^ that may cause endothelial dysfunction and tubulointerstitial damage in transplanted kidneys via chronic inflammation.^39^^,^^40^ Senescent fibroblasts secrete high levels of G-CSF, and IL-1α expression is upregulated in senescent endothelial cells, fibroblasts, and epithelial cells.^4^ MIP-1α is overexpressed by most senescent cells and is also one of the most described chemokines for monocyte and macrophage recruitment during kidney inflammation.^4^^,^^41^

MMPs secreted by senescent cells^4^ are involved in remodeling of the extracellular matrix, and they play a key role in many kidney diseases.^42^ Previous studies on the effect of marine n-3 PUFAs on MMPs have shown conflicting results. Reduced levels of several MMPs have been reported by in vitro experiments, but a similar effect has not been demonstrated in human studies.^17^ Our results showed the mitigating effects of marine n-3 PUFA supplementation on the collagenases MMP-1 and MMP-13 in the plasma, but not on MMP-2, -3, or -9. However, the clinical significance of these findings is uncertain because in vivo regulation of MMP activity using tissue inhibitors of metalloproteinases is complex and not fully understood. Tissue inhibitors of metalloproteinases bind to active and inactive forms of MMPs; thus, measured MMP values may not reflect enzymatic activity levels accurately.^43^

TGF-β1 is involved in the regulation of cell proliferation and differentiation, embryonic development, carcinogenesis, fibrosis, wound healing, angiogenesis, and immune responses.^44^ This regulatory cytokine is highly expressed in the early stage of SASP acquisition.^27^ Upregulation of TGF-β1 promotes kidney fibrosis, and urinary TGF-β1 excretion has been shown to be increased in patients with glomerular disease with and without proteinuria.^45^ In the present study, we did not find a significant difference between the study groups in terms of changes in the urinary TGF-β1 levels during follow-up. However, our assay kit, like most commercially available ones, does not differentiate between latent and active forms of TGF-β1. Because total TGF-β1 is dominated by its latent form, an assay that specifically detects biologically active TGF-β1 might be more appropriate to evaluate treatment effect.^46^

Previous studies of cellular senescence have mainly focused on the SASP secretome in the serum or plasma, and few studies have investigated the urinary excretion of SASP components. We conducted a pilot study in advance to evaluate the effect of matrix interference in urine samples using our assay to get results that are as accurate as possible. Our analyses showed that many of the selected SASP components had levels that were too low to be determined in the urine samples, which can be taken into consideration in future research. We also found no correlations between the plasma and urinary levels of the SASP components (Tables S2 and S3). This may indicate that urinary measurements are not significantly influenced by the glomerular filtration or tubular secretion of circulating plasma components and that urinary SASP components mainly reflect local production from the kidney graft. This supports further investigations of urinary SASP components in future studies of kidney aging.

The baseline plasma phospholipid marine n-3 PUFA levels were quite high in our study population, with a mean content of >6 wt% in both the groups. The beneficial properties of marine n-3 PUFAs may be dose dependent.^17^ Thus, it is possible that the effect of a marine n-3 PUFA intervention might be different in kidney transplant recipients with much lower baseline levels, such as in those in North America.^47^

The strengths of the present study include the analyses of the plasma and urine samples from the intervention group and their comparison with samples from the placebo group, with a relatively high dose of marine n-3 PUFA supplementation, and the plasma fatty acid analysis performed using gold-standard gas chromatography.

This study also has important limitations. Given the exploratory nature of this substudy, our results are largely hypothesis-generating, with data that can inform future studies. Insufficient statistical power due to missing urine samples could possibly explain the lack of a significant effect of marine n-3 PUFA supplementation on the urinary SASP components. Additionally, the SASP components measured in this substudy were not senescence specific. Universal markers with absolute specificity for cellular senescence have not been identified to date.^48^ Some SASP components are commonly featured in senescent cells, regardless of cell type, but the final composition is cell specific and varies considerably depending on the stage of senescence and the induction pathway.^1^^,^^2^^,^^4^

The primary endpoint of the ORENTRA trial was a change in kidney function, defined as mGFR, which was evaluated by measuring iohexol clearance. No significant difference was found between the study groups in terms of changes in mGFR after 44 weeks of follow-up.^24^ In the present study, we found neither any associations between changes in the levels of significant SASP components and mGFR, nor differences in the interstitial fibrosis percentage scores in kidney graft biopsies. Thus, we cannot infer any clinical significance from our findings. Recent recommendations support the use of multiple methods for senescence identification.^49^ Combining the results of the present study with an evaluation of cellular senescence markers in kidney allograft biopsies, such as p16^INK4a^ staining, might further contribute to our understanding of the possible clinical relevance of our findings.

The kidney transplant recipients in our study cohort were enrolled only 8 weeks after transplantation and were, thus, more susceptible to transplantation-related complications. Although the adverse events were evenly distributed between the study groups, there were considerable numbers of bacterial and viral infections, acute graft rejections, and incidental malignancies during follow-up,^24^ all of which might have affected the levels of the measured SASP components. Studies with a transplant cohort in maintenance phase could have provided different results. The ongoing "the long-term effect of marine n-3 polyunsaturated fatty acid supplementation on glomerular filtration rate and development of fibrosis in the renal allograft (EMIRA)" trial (ClinicalTrials.gov identifier NCT03018041) with kidney transplant recipients on maintenance immunosuppression might produce a new insight.

In conclusion, 44 weeks of marine n-3 PUFA supplementation was associated with mitigating effects on the plasma SASP components G-CSF, IL-1α, MIP-1α, MMP-1, and MMP-13 in kidney transplant recipients. We found no associations between changes in the levels of the SASP components and changes in kidney transplant function. Future studies with kidney transplant recipients in maintenance phase, combined with an evaluation of cellular senescence markers in kidney transplant biopsies, are needed to further elucidate the effect of the marine n-3 PUFA on cellular senescence and its potential clinical application in kidney transplant recipients.
