# Supplementary material for: Marine n-3 Polyunsaturated Fatty Acids and Cellular Senescence Markers in Incident Kidney Transplant Recipients: The Omega-3 Fatty Acids in Renal Transplantation (ORENTRA) Randomized Clinical Trial
Source: Kidney Med. 2021 Oct 4;3(6):1041–9. doi: 10.1016/j.xkme.2021.07.010 (PMC8664741; doi:10.1016/j.xkme.2021.07.010)
Supplement: Supplementary File (PDF) — Tables S1-S4. [file mmc1.pdf]

Table S1. Pilot-study of urine sample dilution factors.

	Dilution	Patient A	Patient B	Patient C	Patient D
Urine G-CSF	1:1	26.16	28.69	24.85	20.32
	1:2	58.35	*24.81	35.63	*19.59
	1:5	108.97	117.44	140.96	*46.60
	1:10	184.58	234.89	240.38	168.18
	1:20	475.28	424.21	507.58	362.75
Urine GM-CSF	1:1	Undetectable	Undetectable	Undetectable	Undetectable
	1:2	Undetectable	Undetectable	Undetectable	Undetectable
	1:5	Undetectable	Undetectable	Undetectable	Undetectable
	1:10	Undetectable	Undetectable	Undetectable	Undetectable
	1:20	Undetectable	Undetectable	Undetectable	Undetectable
Urine GROα	1:1	*1.18	2.27	*1.21	2.57
	1:2	*2.35	*3.59	*2.53	6.96
	1:5	*4.84	*6.01	*4.64	10.8
	1:10	*11.38	*10.61	*8.33	*13.50
	1:20	*21.22	*20.44	*14.10	*20.96
Urine IL-1α	1:1	Undetectable	Undetectable	*0.21	3.65
	1:2	Undetectable	*0.72	*0.28	4.95
	1:5	Undetectable	Undetectable	Undetectable	5.54
	1:10	Undetectable	Undetectable	Undetectable	6.39
	1:20	Undetectable	Undetectable	Undetectable	*4.55
Urine IL-1β	1:1	*0.66	*0.59	1.56	2.56
	1:2	*2.38	*2.08	*0.20	*2.38
	1:5	*0.93	*0.50	*1.35	8.87
	1:10	*3.51	Undetectable	Undetectable	Undetectable
	1:20	Undetectable	Undetectable	Undetectable	*8.62
Urine IL-6	1:1	31.34	35.69	30.7	34.88
	1:2	56.99	65.22	54.4	69.21
	1:5	133.22	77.08	108.85	123.44
	1:10	188.13	99.05	107.23	204.41
	1:20	288.62	162.83	*128.90	383.95
Urine IL-8	1:1	2.56	12.66	8.38	38.32
	1:2	*4.30	18.25	10.67	69.72
	1:5	*5.32	22.59	*10.68	78.28
	1:10	*6.76	24.1	*5.77	96.39
	1:20	*2.52	*27.29	OOR <	94.76

Urine MCP-1	1:1	152.75	835.12	411.88	516.53
	1:2	230.95	1041.02	455.17	881.99
	1:5	233.69	1392.23	472.75	983.75
	1:10	253.45	1363.54	409.2	1081.15
	1:20	234.98	1209.5	380.1	898.41
Urine MCP-2	1:1	*0.21	1.04	*0.03	3.66
	1:2	*0.45	1.27	*0.12	4.64
	1:5	*0.16	*0.88	Undetectable	5.05
	1:10	Undetectable	Undetectable	Undetectable	*3.32
	1:20	Undetectable	Undetectable	Undetectable	*2.65
Urine MCP-3	1:1	21.36	15.52	18.15	*13.63
	1:2	*25.26	29.19	*23.16	32.85
	1:5	*52.39	*25.69	*25.69	*57.90
	1:10	*104.79	*51.39	*51.39	*66.85
	1:20	*161.12	*209.58	Undetectable	*102.77
Urine MIP-1α	1:1	*1.32	*1.32	*1.35	*1.54
	1:2	*2.75	*1.74	*1.04	*2.75
	1:5	*3.34	Undetectable	Undetectable	*3.52
	1:10	*3.50	Undetectable	Undetectable	*1.99
	1:20	*1.32	Undetectable	Undetectable	Undetectable
Urine MMP-1	1:1	Undetectable	*1.83	Undetectable	Undetectable
	1:2	Undetectable	*3.36	Undetectable	Undetectable
	1:5	Undetectable	Undetectable	Undetectable	Undetectable
	1:10	Undetectable	Undetectable	Undetectable	Undetectable
	1:20	Undetectable	Undetectable	Undetectable	Undetectable
Urine PAI-1	1:1	*5.30	38.31	*10.96	*19.34
	1:2	*8.59	67.78	*10.59	*23.74
	1:5		*36.17	Undetectable	*31.36
	1:10	Undetectable	Undetectable	Undetectable	Undetectable
	1:20	Undetectable	Undetectable	Undetectable	Undetectable
Urine TNFα	1:1	18.76	13.49	19.53	23.99
	1:2	33.58	40.59	35.17	38.29
	1:5	60.94	*44.88	*29.61	103.35
	1:10	*89.77	*22.48	Undetectable	99.25
	1:20	Undetectable	Undetectable	*10.99	*179.54

Variables shown as pg/ml.
* below the range of the standard curve, estimated values.
Undetectable: Beneath the detection limit of the assay.

G-CSF, granulocyte colony-stimulating factor; GM-CSF, granulocyte-macrophage colony-stimulating factor; GROα, growth-regulated oncogene α; IL-1α, interleukin 1α; IL-1β, interleukin 1β; IL-6, interleukin 6; IL-8, interleukin 8; MCP-1, monocyte chemoattractant protein-1; MCP-2, monocyte chemoattractant protein-2; MCP-3, monocyte chemoattractant protein-3; MIP-1α, Macrophage inflammatory protein 1α; MMP-1, matrix metalloproteinase-1; PAI-1, plasminogen activator inhibitor-1; TNFα, Tumor necrosis factor α.

Table S2. Correlation between plasma and urine values at baseline.

		BASELINE					
		Plasma G-CSF	Plasma IL-6	Plasma IL-8	Plasma MCP-1	Plasma MMP-3	Plasma MMP-9
Urine G-CSF	Correlation Coefficient.	0.075					
	Sig. (2-tailed)	0.42					
	N	120					
Urine IL-6	Correlation Coefficient		-0.033				
	Sig. (2-tailed)		0.72				
	N		120				
Urine IL-8	Correlation Coefficient			-0.042			
	Sig. (2-tailed)			0.65			
	N			120			
Urine MCP-1	Correlation Coefficient				0.022		
	Sig. (2-tailed)				0.81		
	N				120		
Urine MMP-3	Correlation Coefficient					0.061	
	Sig. (2-tailed)					0.51	
	N					120	
Urine MMP-9	Correlation Coefficient						0.025
	Sig. (2-tailed)						0.79
	N						120

Associations between plasma (in pg/ml) and the corresponding urine variables (in ng/g creatinine) at baseline, assessed by Pearson's correlation coefficient (r).

G-CSF, granulocyte colony-stimulating factor; IL-6, interleukin 6; IL-8, interleukin 8; MCP-1, monocyte chemoattractant protein-1; MMP-3, matrix metalloproteinase-3; MMP-9, matrix metalloproteinase-9.

Table S3. Correlation between plasma and urine values at end of study.

**END OF STUDY**							
		Plasma G-CSF	Plasma IL-6	Plasma IL-8	Plasma MCP-1	Plasma MMP-3	Plasma MMP-9
Urine G-CSF	Correlation Coefficient.	-0.032					
	Sig. (2-tailed)	0.80					
	N	65					
Urine IL-6	Correlation Coefficient		0.029				
	Sig. (2-tailed)		0.82				
	N		65				
Urine IL-8	Correlation Coefficient			0.021			
	Sig. (2-tailed)			0.87			
	N			65			
Urine MCP-1	Correlation Coefficient				0.198		
	Sig. (2-tailed)				0.11		
	N				65		
Urine MMP-3	Correlation Coefficient					0.058	
	Sig. (2-tailed)					0.65	
	N					65	
Urine MMP-9	Correlation Coefficient						-0.018
	Sig. (2-tailed)						0.89
	N						65

Associations between plasma (in pg/ml) and the corresponding urine variables (in ng/g creatinine) at end of study, assessed by Pearson's correlation coefficient (r).

G-CSF, granulocyte colony-stimulating factor; IL-6, interleukin 6; IL-8, interleukin 8; MCP-1, monocyte chemoattractant protein-1; MMP-3, matrix metalloproteinase-3; MMP-9, matrix metalloproteinase-9.

Table S4. Correlation between changes in levels of significant SASP components, measured glomerular filtration rate (mGFR) and interstitial fibrosis percentage (IF%) score.

		Δ Measured GFR (ml/min)
Δ Plasma G-CSF	Correlation Coefficient.	-0.051
	Sig. (2-tailed)	0.58
	N	118
Δ Plasma IL-1α	Correlation Coefficient	-0.147
	Sig. (2-tailed)	0.11
	N	118
Δ Plasma MIP-1α	Correlation Coefficient	-0.100
	Sig. (2-tailed)	0.28
	N	117
Δ Plasma MMP-1	Correlation Coefficient	-0.170
	Sig. (2-tailed)	0.07
	N	118
Δ Plasma MMP-13	Correlation Coefficient	-0.129
	Sig. (2-tailed)	0.16
	N	118

		Δ IF% Score
Δ Plasma G-CSF	Correlation Coefficient.	0.113
	Sig. (2-tailed)	0.26
	N	118
Δ Plasma IL-1α	Correlation Coefficient	0.081
	Sig. (2-tailed)	0.42
	N	118
Δ Plasma MIP-1α	Correlation Coefficient	-0.058
	Sig. (2-tailed)	0.57
	N	117
Δ Plasma MMP-1	Correlation Coefficient	0.162
	Sig. (2-tailed)	0.11
	N	118
Δ Plasma MMP-13	Correlation Coefficient	-0.187
	Sig. (2-tailed)	0.06
	N	118

Associations assessed by Pearson's correlation coefficient (r).

G-CSF, granulocyte colony-stimulating factor; IL-1α, interleukin 1α; MIP-1α, macrophage inflammatory protein 1α; MMP-1, matrix metalloproteinase 1; MMP-13, matrix metalloproteinase 13.
